# Dichloridobis[1-(2-methyl­benzimidazol-1-ylmethyl-κ*N* ^3^)benzotriazole]mercury(II)

**DOI:** 10.1107/S1600536809023459

**Published:** 2009-06-27

**Authors:** Jie Wu, Jie Yang, Fang-fang Pan

**Affiliations:** a Department of Chemistry, Zhengzhou University, Zhengzhou 450052, People’s Republic of China

## Abstract

In the title compound, [HgCl_2_(C_15_H_13_N_5_)_2_], the Hg^II^ atom is located on a twofold rotation axis and resides in a distorted tetra­hedral coordination environment composed of two Cl atoms and two N atoms from two 1-(2-methyl­benzimidazol-1-ylmeth­yl)benzotriazole ligands.

## Related literature

For metal complexes of similar *N*-heterocyclic ligands, see: Fan *et al.* (2003 ▶); Hoskins *et al.* (1997 ▶); Makoto *et al.* (2005 ▶)
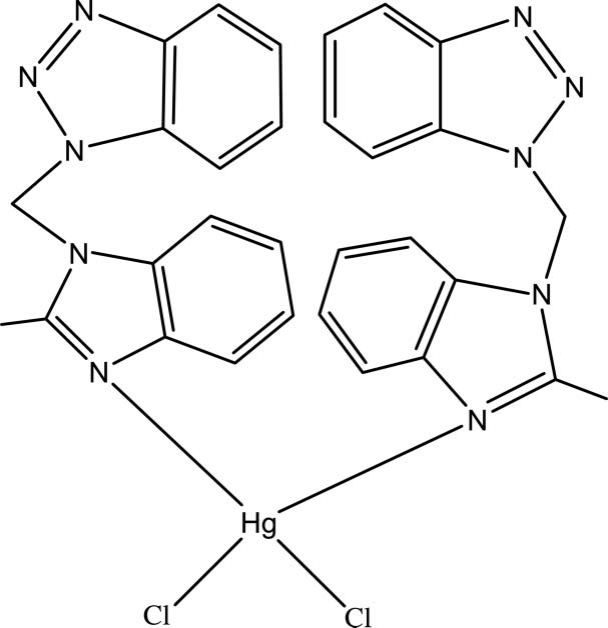


## Experimental

### 

#### Crystal data

[HgCl_2_(C_15_H_13_N_5_)_2_]
*M* *_r_* = 798.10
Monoclinic, 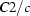

*a* = 15.612 (3) Å
*b* = 12.883 (3) Å
*c* = 14.751 (3) Å
β = 97.49 (3)°
*V* = 2941.5 (11) Å^3^
*Z* = 4
Mo *K*α radiation
μ = 5.46 mm^−1^
*T* = 293 K
0.22 × 0.18 × 0.16 mm

#### Data collection

Rigaku Saturn724 diffractometer
Absorption correction: multi-scan (*CrystalClear*; Rigaku/MSC, 2006 ▶) *T*_min_ = 0.380, *T*_max_ = 0.476 (expected range = 0.334–0.418)
14609 measured reflections
2587 independent reflections
2379 reflections with *I* > 2σ(*I*)
*R*_int_ = 0.051

#### Refinement

*R*[*F* ^2^ > 2σ(*F* ^2^)] = 0.036
*wR*(*F* ^2^) = 0.062
*S* = 1.09
2587 reflections
196 parameters
H-atom parameters constrained
Δρ_max_ = 0.57 e Å^−3^
Δρ_min_ = −0.47 e Å^−3^

### 

Data collection: *CrystalClear* (Rigaku/MSC, 2006 ▶); cell refinement: *CrystalClear*; data reduction: *CrystalClear*; program(s) used to solve structure: *SHELXS97* (Sheldrick, 2008 ▶); program(s) used to refine structure: *SHELXS97* (Sheldrick, 2008 ▶); molecular graphics: *SHELXTL* (Sheldrick, 2008 ▶); software used to prepare material for publication: *SHELXTL*.

## Supplementary Material

Crystal structure: contains datablocks global, I. DOI: 10.1107/S1600536809023459/ng2600sup1.cif

Structure factors: contains datablocks I. DOI: 10.1107/S1600536809023459/ng2600Isup2.hkl

Additional supplementary materials:  crystallographic information; 3D view; checkCIF report

## Figures and Tables

**Fig. 1.**
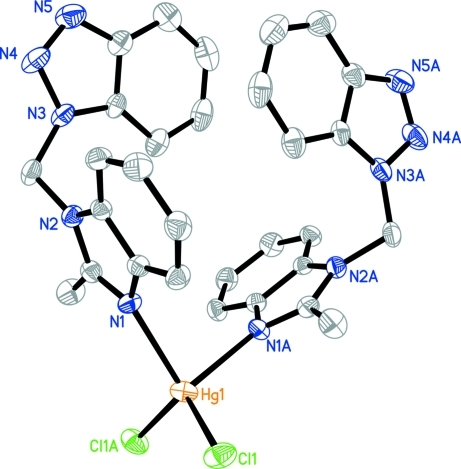
View of the title complex, showing the labeling of the non-H atoms and 30% probability ellipsolids.

## supplementary crystallographic information

### Comment

The complexation of metal ions by nitrogen heterocyclic compounds has been extensively studied. Owing to the unique ability of the heterocyclic compounds to form stable chelates with various coordiantion modes and its biological activity, many crystal ctructures have been determined (Fan, *et al.*, 2003; Hoskins, *et al.* 1997; Makoto,*et al.*,2005). *N*-(2-methyl-benzoimidazol-3-yl-methyl)-benzotriazole, has the benzotriazole group and the benzoimidazole group and can offer possibilities to form complicated coordiantion compounds. However, the coordiantion chemistry and structural properties of metal complexes with the ligand has never been documented to data. In this paper, we reported the synthesis and crystal structure of the title compound, (I). In (I) (Fig. 1), the HgII atom is coordinated by two Cl atoms and two N atoms from the ligand to form a distorted tetrahedral coordination environment. Each ligand is coordianted to the Hg atom in a monodentate fashion. In the ligand, the benzotriazole group and benzotriazole group is bridged by a methylene, with an N—C—N angle of 111.3 (4)°. The benzotriazole group and the benzoimidazole group are almost perpendicular with each other, with the dihedral angle being 89.9°. Thus, two ligands are bridged by the Hg atom to form a cage-like compound.

### Experimental

The ligand *N*-(2-methyl-benzoimidazol-3-yl-methyl)-benzotriazole (0.04 mmol, 0.118 g) in MeOH (6 ml) was added dropwise to a solution of HgCl2 (0.4 mmol, 0.108 g) in methanol (3 ml). The precipitate was filtered and the resulting solution was allowed to stand at room temperature in the dark. After one week good quality colorless crystals were obtained from the filtrate and dried in air.

### Refinement

H atoms were generated geometrically, with C-H = 0.96,  0.86 and 0.93Å for methyl, N and aromatic H, respectively, and constrained to ride their parent atoms with Uiso(H) = x times Ueq(C), where x = 1.5 for methyl H and x = 1.2 for all other H atoms.

### Crystal data

**Table:**

[HgCl_2_(C_15_H_13_N_5_)_2_]	*F*(000) = 1560
*M**_r_* = 798.10	*D*_x_ = 1.802 Mg m^−^^3^
Monoclinic, *C*2/*c*	Mo *K*α radiation, λ = 0.71073 Å
Hall symbol: -C 2yc	Cell parameters from 4239 reflections
*a* = 15.612 (3) Å	θ = 2.1–29.1°
*b* = 12.883 (3) Å	µ = 5.46 mm^−^^1^
*c* = 14.751 (3) Å	*T* = 293 K
β = 97.49 (3)°	Prism, colorless
*V* = 2941.5 (11) Å^3^	0.22 × 0.18 × 0.16 mm
*Z* = 4	

### Data collection

**Table:**

Rigaku Saturn724 diffractometer	2587 independent reflections
Radiation source: fine-focus sealed tube	2379 reflections with *I* > 2σ(*I*)
graphite	*R*_int_ = 0.051
Detector resolution: 28.5714 pixels mm^-1^	θ_max_ = 25.0°, θ_min_ = 2.4°
dtprofit.ref scans	*h* = −18→18
Absorption correction: multi-scan (*CrystalClear*; Rigaku/MSC, 2006)	*k* = −15→15
*T*_min_ = 0.380, *T*_max_ = 0.476	*l* = −17→17
14609 measured reflections	

### Refinement

**Table:**

Refinement on *F*^2^	Primary atom site location: structure-invariant direct methods
Least-squares matrix: full	Secondary atom site location: difference Fourier map
*R*[*F*^2^ > 2σ(*F*^2^)] = 0.036	Hydrogen site location: inferred from neighbouring sites
*wR*(*F*^2^) = 0.062	H-atom parameters constrained
*S* = 1.09	*w* = 1/[σ^2^(*F*_o_^2^) + (0.0229*P*)^2^ + 4.6614*P*] where *P* = (*F*_o_^2^ + 2*F*_c_^2^)/3
2587 reflections	(Δ/σ)_max_ < 0.001
196 parameters	Δρ_max_ = 0.57 e Å^−^^3^
0 restraints	Δρ_min_ = −0.47 e Å^−^^3^

### Special details

**Table:**

Geometry. All e.s.d.'s (except the e.s.d. in the dihedral angle between two l.s. planes) are estimated using the full covariance matrix. The cell e.s.d.'s are taken into account individually in the estimation of e.s.d.'s in distances, angles and torsion angles; correlations between e.s.d.'s in cell parameters are only used when they are defined by crystal symmetry. An approximate (isotropic) treatment of cell e.s.d.'s is used for estimating e.s.d.'s involving l.s. planes.
Refinement. Refinement of *F*^2^ against ALL reflections. The weighted *R*-factor *wR* and goodness of fit *S* are based on *F*^2^, conventional *R*-factors *R* are based on *F*, with *F* set to zero for negative *F*^2^. The threshold expression of *F*^2^ > σ(*F*^2^) is used only for calculating *R*-factors(gt) *etc*. and is not relevant to the choice of reflections for refinement. *R*-factors based on *F*^2^ are statistically about twice as large as those based on *F*, and *R*- factors based on ALL data will be even larger.

### Fractional atomic coordinates and isotropic or equivalent isotropic displacement parameters (Å^2^)

**Table:**

	*x*	*y*	*z*	*U*_iso_*/*U*_eq_	
Hg1	0.5000	−0.10389 (2)	0.2500	0.04638 (11)	
Cl1	0.62989 (9)	−0.19849 (10)	0.30815 (8)	0.0585 (4)	
N1	0.4757 (2)	0.0231 (3)	0.3545 (2)	0.0389 (9)	
N2	0.4113 (2)	0.1501 (3)	0.4213 (2)	0.0380 (9)	
N3	0.3561 (2)	0.3232 (3)	0.4176 (2)	0.0439 (10)	
N4	0.3539 (3)	0.3999 (4)	0.4808 (3)	0.0606 (12)	
N5	0.3651 (3)	0.4882 (4)	0.4425 (3)	0.0642 (13)	
C1	0.3170 (3)	0.0391 (4)	0.3110 (4)	0.0577 (14)	
H1A	0.3243	−0.0205	0.2737	0.087*	
H1B	0.2941	0.0956	0.2728	0.087*	
H1C	0.2778	0.0226	0.3538	0.087*	
C2	0.4021 (3)	0.0696 (4)	0.3614 (3)	0.0392 (11)	
C3	0.5386 (3)	0.0762 (3)	0.4139 (3)	0.0359 (11)	
C4	0.6271 (3)	0.0600 (4)	0.4337 (3)	0.0436 (11)	
H4	0.6547	0.0066	0.4064	0.052*	
C5	0.6722 (3)	0.1269 (4)	0.4956 (3)	0.0513 (13)	
H5	0.7317	0.1191	0.5095	0.062*	
C6	0.6311 (3)	0.2054 (4)	0.5377 (3)	0.0541 (13)	
H6	0.6637	0.2481	0.5799	0.065*	
C7	0.5436 (3)	0.2220 (4)	0.5189 (3)	0.0452 (12)	
H7	0.5163	0.2750	0.5469	0.054*	
C8	0.4984 (3)	0.1560 (3)	0.4564 (3)	0.0348 (10)	
C9	0.3430 (3)	0.2172 (4)	0.4447 (3)	0.0471 (12)	
H9A	0.2878	0.1926	0.4145	0.057*	
H9B	0.3414	0.2146	0.5102	0.057*	
C10	0.3696 (3)	0.3647 (4)	0.3355 (3)	0.0420 (11)	
C11	0.3763 (3)	0.3232 (5)	0.2497 (3)	0.0568 (14)	
H11	0.3719	0.2523	0.2381	0.068*	
C12	0.3900 (3)	0.3936 (5)	0.1833 (4)	0.0636 (16)	
H12	0.3946	0.3697	0.1246	0.076*	
C13	0.3973 (3)	0.5008 (6)	0.2009 (4)	0.0688 (17)	
H13	0.4074	0.5456	0.1539	0.083*	
C14	0.3898 (3)	0.5404 (5)	0.2845 (4)	0.0642 (16)	
H14	0.3943	0.6114	0.2959	0.077*	
C15	0.3752 (3)	0.4707 (4)	0.3524 (3)	0.0496 (13)	

### Atomic displacement parameters (Å^2^)

**Table:**

	*U*^11^	*U*^22^	*U*^33^	*U*^12^	*U*^13^	*U*^23^
Hg1	0.05294 (19)	0.03316 (15)	0.05032 (18)	0.000	−0.00348 (13)	0.000
Cl1	0.0714 (9)	0.0507 (8)	0.0509 (7)	0.0232 (7)	−0.0012 (7)	0.0044 (6)
N1	0.039 (2)	0.034 (2)	0.044 (2)	0.0053 (18)	0.0057 (18)	−0.0056 (17)
N2	0.035 (2)	0.039 (2)	0.040 (2)	0.0014 (17)	0.0063 (18)	−0.0022 (18)
N3	0.050 (2)	0.045 (2)	0.037 (2)	0.0158 (19)	0.0079 (19)	−0.0040 (19)
N4	0.080 (3)	0.058 (3)	0.045 (2)	0.023 (3)	0.011 (2)	−0.010 (2)
N5	0.085 (4)	0.048 (3)	0.060 (3)	0.014 (3)	0.006 (3)	−0.003 (2)
C1	0.042 (3)	0.063 (4)	0.064 (3)	−0.001 (3)	−0.005 (3)	−0.013 (3)
C2	0.040 (3)	0.040 (3)	0.038 (3)	−0.005 (2)	0.004 (2)	0.001 (2)
C3	0.041 (3)	0.034 (2)	0.032 (2)	−0.002 (2)	0.003 (2)	−0.0002 (19)
C4	0.036 (3)	0.046 (3)	0.049 (3)	0.004 (2)	0.007 (2)	−0.004 (2)
C5	0.038 (3)	0.057 (3)	0.058 (3)	−0.006 (2)	0.005 (2)	−0.001 (3)
C6	0.054 (3)	0.057 (3)	0.049 (3)	−0.010 (3)	−0.001 (3)	−0.010 (3)
C7	0.051 (3)	0.039 (3)	0.046 (3)	0.001 (2)	0.007 (2)	−0.006 (2)
C8	0.040 (3)	0.032 (2)	0.032 (2)	−0.001 (2)	0.005 (2)	0.001 (2)
C9	0.044 (3)	0.056 (3)	0.045 (3)	0.012 (2)	0.018 (2)	0.000 (2)
C10	0.034 (3)	0.054 (3)	0.039 (3)	0.009 (2)	0.007 (2)	−0.001 (2)
C11	0.053 (3)	0.074 (4)	0.043 (3)	0.009 (3)	0.007 (3)	−0.004 (3)
C12	0.047 (3)	0.107 (5)	0.036 (3)	0.005 (3)	0.004 (2)	−0.002 (3)
C13	0.043 (3)	0.094 (5)	0.068 (4)	0.001 (3)	0.001 (3)	0.033 (4)
C14	0.050 (3)	0.064 (4)	0.076 (4)	0.006 (3)	−0.001 (3)	0.016 (3)
C15	0.047 (3)	0.054 (3)	0.047 (3)	0.014 (3)	0.002 (2)	0.003 (3)

### Geometric parameters (Å, °)

**Table:**

Hg1—N1	2.313 (3)	C4—C5	1.380 (7)
Hg1—N1^i^	2.313 (3)	C4—H4	0.9300
Hg1—Cl1^i^	2.4248 (13)	C5—C6	1.387 (7)
Hg1—Cl1	2.4248 (13)	C5—H5	0.9300
N1—C2	1.311 (5)	C6—C7	1.375 (6)
N1—C3	1.405 (5)	C6—H6	0.9300
N2—C2	1.357 (6)	C7—C8	1.378 (6)
N2—C8	1.392 (5)	C7—H7	0.9300
N2—C9	1.450 (5)	C9—H9A	0.9700
N3—N4	1.362 (5)	C9—H9B	0.9700
N3—C10	1.366 (6)	C10—C15	1.389 (7)
N3—C9	1.444 (6)	C10—C11	1.390 (6)
N4—N5	1.293 (6)	C11—C12	1.372 (7)
N5—C15	1.377 (6)	C11—H11	0.9300
C1—C2	1.488 (6)	C12—C13	1.407 (8)
C1—H1A	0.9600	C12—H12	0.9300
C1—H1B	0.9600	C13—C14	1.354 (8)
C1—H1C	0.9600	C13—H13	0.9300
C3—C4	1.391 (6)	C14—C15	1.387 (7)
C3—C8	1.395 (6)	C14—H14	0.9300
			
N1—Hg1—N1^i^	89.97 (18)	C6—C5—H5	119.1
N1—Hg1—Cl1^i^	112.90 (10)	C7—C6—C5	121.8 (5)
N1^i^—Hg1—Cl1^i^	108.78 (10)	C7—C6—H6	119.1
N1—Hg1—Cl1	108.78 (10)	C5—C6—H6	119.1
N1^i^—Hg1—Cl1	112.90 (10)	C6—C7—C8	116.6 (4)
Cl1^i^—Hg1—Cl1	119.65 (7)	C6—C7—H7	121.7
C2—N1—C3	106.1 (4)	C8—C7—H7	121.7
C2—N1—Hg1	126.6 (3)	C7—C8—N2	132.3 (4)
C3—N1—Hg1	126.7 (3)	C7—C8—C3	122.3 (4)
C2—N2—C8	107.5 (4)	N2—C8—C3	105.4 (4)
C2—N2—C9	126.3 (4)	N3—C9—N2	111.3 (4)
C8—N2—C9	126.2 (4)	N3—C9—H9A	109.4
N4—N3—C10	110.1 (4)	N2—C9—H9A	109.4
N4—N3—C9	118.7 (4)	N3—C9—H9B	109.4
C10—N3—C9	131.2 (4)	N2—C9—H9B	109.4
N5—N4—N3	108.8 (4)	H9A—C9—H9B	108.0
N4—N5—C15	108.4 (4)	N3—C10—C15	103.8 (4)
C2—C1—H1A	109.5	N3—C10—C11	134.1 (5)
C2—C1—H1B	109.5	C15—C10—C11	122.1 (5)
H1A—C1—H1B	109.5	C12—C11—C10	115.7 (5)
C2—C1—H1C	109.5	C12—C11—H11	122.2
H1A—C1—H1C	109.5	C10—C11—H11	122.2
H1B—C1—H1C	109.5	C11—C12—C13	122.2 (5)
N1—C2—N2	112.3 (4)	C11—C12—H12	118.9
N1—C2—C1	125.1 (4)	C13—C12—H12	118.9
N2—C2—C1	122.6 (4)	C14—C13—C12	121.6 (5)
C4—C3—C8	120.6 (4)	C14—C13—H13	119.2
C4—C3—N1	130.7 (4)	C12—C13—H13	119.2
C8—C3—N1	108.7 (4)	C13—C14—C15	117.1 (6)
C5—C4—C3	116.9 (4)	C13—C14—H14	121.4
C5—C4—H4	121.6	C15—C14—H14	121.4
C3—C4—H4	121.6	N5—C15—C14	129.9 (5)
C4—C5—C6	121.8 (5)	N5—C15—C10	108.8 (4)
C4—C5—H5	119.1	C14—C15—C10	121.3 (5)
			
N1^i^—Hg1—N1—C2	86.5 (4)	C9—N2—C8—C7	−0.3 (8)
Cl1^i^—Hg1—N1—C2	−24.0 (4)	C2—N2—C8—C3	0.3 (5)
Cl1—Hg1—N1—C2	−159.3 (3)	C9—N2—C8—C3	−180.0 (4)
N1^i^—Hg1—N1—C3	−83.5 (3)	C4—C3—C8—C7	0.2 (7)
Cl1^i^—Hg1—N1—C3	166.0 (3)	N1—C3—C8—C7	179.9 (4)
Cl1—Hg1—N1—C3	30.7 (4)	C4—C3—C8—N2	179.9 (4)
C10—N3—N4—N5	0.3 (6)	N1—C3—C8—N2	−0.3 (5)
C9—N3—N4—N5	−178.6 (4)	N4—N3—C9—N2	−128.3 (4)
N3—N4—N5—C15	−0.3 (6)	C10—N3—C9—N2	53.1 (7)
C3—N1—C2—N2	0.0 (5)	C2—N2—C9—N3	−116.4 (5)
Hg1—N1—C2—N2	−171.7 (3)	C8—N2—C9—N3	63.9 (6)
C3—N1—C2—C1	−179.6 (4)	N4—N3—C10—C15	−0.2 (5)
Hg1—N1—C2—C1	8.7 (7)	C9—N3—C10—C15	178.5 (5)
C8—N2—C2—N1	−0.2 (5)	N4—N3—C10—C11	−179.2 (5)
C9—N2—C2—N1	−179.9 (4)	C9—N3—C10—C11	−0.4 (9)
C8—N2—C2—C1	179.4 (4)	N3—C10—C11—C12	179.7 (5)
C9—N2—C2—C1	−0.3 (7)	C15—C10—C11—C12	0.8 (7)
C2—N1—C3—C4	180.0 (5)	C10—C11—C12—C13	0.3 (8)
Hg1—N1—C3—C4	−8.4 (7)	C11—C12—C13—C14	−1.0 (8)
C2—N1—C3—C8	0.2 (5)	C12—C13—C14—C15	0.4 (8)
Hg1—N1—C3—C8	171.9 (3)	N4—N5—C15—C14	−179.2 (5)
C8—C3—C4—C5	−0.8 (7)	N4—N5—C15—C10	0.1 (6)
N1—C3—C4—C5	179.5 (4)	C13—C14—C15—N5	−180.0 (5)
C3—C4—C5—C6	1.2 (7)	C13—C14—C15—C10	0.8 (8)
C4—C5—C6—C7	−1.1 (8)	N3—C10—C15—N5	0.0 (5)
C5—C6—C7—C8	0.5 (7)	C11—C10—C15—N5	179.2 (4)
C6—C7—C8—N2	−179.7 (5)	N3—C10—C15—C14	179.4 (4)
C6—C7—C8—C3	0.0 (7)	C11—C10—C15—C14	−1.4 (8)
C2—N2—C8—C7	−180.0 (5)		

Symmetry codes: (i) −*x*+1, *y*, −*z*+1/2.
